# Efficacy of Fractional CO_2_ Laser Therapy in Improving Symptoms and Quality of Life in Women with Refractory Vulvar Lichen Sclerosus: A Prospective Observational Study

**DOI:** 10.3390/life14121678

**Published:** 2024-12-18

**Authors:** Ana Gil-Villalba, Ángela Ayén-Rodríguez, María José Naranjo-Díaz, Laura Linares-González, Ricardo Ruiz-Villaverde

**Affiliations:** 1 Dermatology Department, Hospital Universitario Nuestra Señora de Candelaria, 38010 Santa Cruz de Tenerife, Spain; 2 Instituto Biosanitario de Granada, Ibs, 18012 Granada, Spain; angela.ayen.sspa@juntadeandalucia.es (Á.A.-R.); mariaj.naranjo.sspa@juntadeandalucia.es (M.J.N.-D.); laura.linares.sspa@juntadeandalucia.es (L.L.-G.); ricardo.ruiz.villaverde.sspa@juntadeandalucia.es (R.R.-V.); 3 Dermatology Department, Hospital Universitario San Cecilio, 18016 Granada, Spain

**Keywords:** lichen sclerosus, laser therapy, carbon dioxide laser, sexual function, dyspareunia

## Abstract

Lichen sclerosus (LS) is a chronic inflammatory condition predominantly affecting the anogenital region of postmenopausal women. It is associated with considerable aesthetic and functional impairments and an increased risk of squamous cell carcinoma. While high-potency topical corticosteroids remain the cornerstone of treatment, therapeutic options for patients with refractory LS are scarce. Fractional CO_2_ laser therapy has emerged as a potential second-line intervention aiming to mitigate symptoms and improve quality of life. This prospective observational study investigated the short-term efficacy and safety of fractional CO_2_ laser therapy in 75 women with refractory LS who underwent four treatment sessions between January 2022 and February 2024. Sixty-nine patients completed the protocol, demonstrating significant reductions in key symptoms, including pruritus (VAS score from 7.53 ± 3.02 to 4.08 ± 3.07), pain (5.83 ± 3.84 to 2.42 ± 2.85), and dyspareunia (8.26 ± 2.82 to 6.34 ± 3.30). Quality of life, sexual function, and psychological well-being also improved, as evidenced by reductions in Dermatology Life Quality Index (DLQI) scores (10.72 ± 7.25 to 5.94 ± 5.16), enhancements in sexual function (FSFI scores from 10.48 ± 8.46 to 15.52 ± 9.59), and decreased depression severity (BDI scores from 16.66 ± 12.64 to 5.94 ± 5.16). Importantly, no adverse effects were reported during the study period. Although these findings highlight the potential of fractional CO_2_ laser therapy as a safe and effective adjunct for refractory LS, it is essential to acknowledge the study’s limitations, particularly the relatively short follow-up period. Longer-term studies are warranted to confirm sustained benefits and to evaluate the broader applicability of this approach.

## 1. Introduction

Lichen sclerosus (LS) is a chronic inflammatory condition that predominantly involves the anogenital region. It exhibits a higher prevalence among women than in men, with reported female-to-male ratios ranging from 3:1 to 10:1, and is most frequently observed in postmenopausal women. The estimated incidence of LS is 0.1–0.3% in the general population, increasing to 1.6% in women aged over 80 years.

It is a multifactorial disease with both autoimmune and environmental triggers. Several studies suggest an association between LS and autoimmune conditions, particularly systemic lupus erythematosus, Sjögren syndrome, atopic dermatitis, vitiligo, alopecia, and morphea, likely due to shared pathophysiological mechanisms. In contrast, conditions such as psoriasis and type 1 diabetes show reduced odds of association with LS. Thyroid-related autoimmune diseases, including hypothyroidism, Hashimoto thyroiditis, and Graves disease, have been reported at increased rates in LS patients in some large studies, though no significant differences were observed in others. Autoimmune gastrointestinal diseases, such as Crohn’s disease and celiac disease, also appear to increase LS risk, while ulcerative colitis shows an inverse association. Physical trauma and chronic irritation have also been implicated as environmental triggers. Genital injuries, particularly from childbirth, may predispose parous women to LS, with the risk being highest after a single childbirth. Chronic irritation from urinary incontinence, both stress and urge types, has similarly been linked to LS, although evidence from meta-analyses remains inconclusive. Interestingly, certain comorbidities and lifestyle factors show a reduced association with LS. Coronary artery disease, hypertension, diabetes, and myocardial infarction are inversely related to LS risk, possibly reflecting distinct inflammatory pathways. Additionally, LS risk does not appear to be influenced by socioeconomic status or place of residence. These findings highlight the complex interplay of genetic, immune-mediated, and environmental factors in the development of LS, underscoring the need for further research to elucidate its etiology and optimize management strategies [1,2,3,4]. 

**Figure 1:**
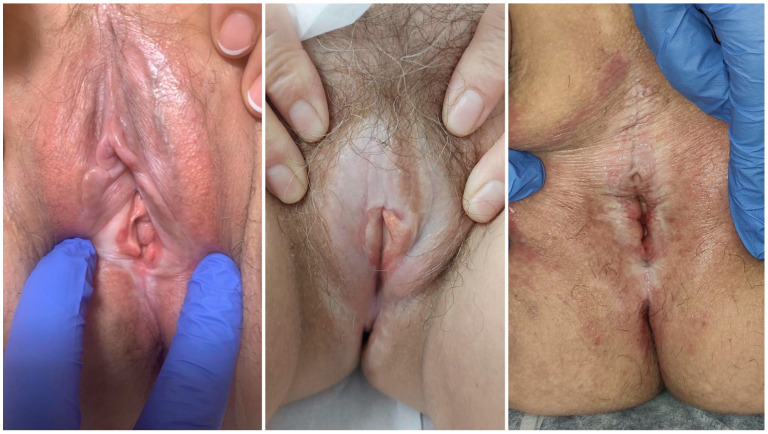
Clinical manifestations of vLS in different stages of evolution. Atrophic whitish plaques on labia minora, inner surface of labia majora, and perineum. Reabsorption of labia minora, burial of the clitoris, and narrowing of the introitus.

Vulvar LS (vLS) lesions are ivory-colored, atrophic plaques, sometimes with erythema, ecchymosis, and fissures (Figure 1). Affected areas often include the labia minora, inner labia majora, clitoral hood, perineum, and perianal region, giving a characteristic “figure-eight” pattern. Long-term scarring leads to loss of vulvar architecture. Common symptoms include pruritus, soreness, dyspareunia, and anal discomfort, significantly impacting quality of life (QoL) and sexual well-being. However, up to 30% of patients remain asymptomatic [1,2,3]. Additional manifestations should be monitored over time, such as dysuria, bladder pain (abacterial cystitis), and obstructive lower urinary tract symptoms (LUTS). Untreated LS carries a 4–6.7% risk of developing vulvar squamous cell carcinoma (vSCC) [5]. 

Extragenital involvement (eLS) occurs in 15–20% of patients, often simultaneously with vLS. Only 6% of cases appear in isolation. eLS presents as asymptomatic morphea-like plaques. Typical locations include breasts, back, shoulders, and thighs. Oral LS (oLS) is extremely rare; clinically, it is similar to oral lichen planus (LP), with whitish papules or plaques on the labial or palatal mucosa. Lip lesions in LS exhibit a vitiligo-like appearance [6,7].

Diagnosis is primarily clinical; the role of biopsy in LS remains controversial. Histology shows hyperkeratosis, epidermal atrophy with loss of rete ridges, and upper dermal changes, such as homogenized, hyalinized collagen bundles with a lichenoid inflammatory infiltrate. However, histological findings may be nonspecific and a strong clinicopathologic correlation is needed [8,9].

Prior to initiating any treatment, it is crucial to document the patient’s baseline status. Photographic documentation during the initial consultation facilitates objective monitoring of disease progression. Symptom severity should be assessed using numerical rating scales (NRS), while quality of life and sexual function should be evaluated through the use of validated questionnaires. These parameters should be re-assessed during follow-up visits. Initial management should prioritize basic care, including appropriate genital hygiene without excessive washing, and the avoidance of potential triggers, such as tight clothing. Preference should be given to loose-fitting garments made from cotton or silk. Fragrance-free emollients should be applied regularly to serve as barrier ointments, protecting against external irritants such as urine, toilet paper, and friction during sexual activity [10].

Lichen sclerosus (LS) is a chronic inflammatory disorder with a recognized risk of malignant progression, particularly to vulvar squamous cell carcinoma (SCC), through a precursor lesion known as differentiated vulvar intraepithelial neoplasia (dVIN). The malignancy potential of vulvar LS (VLS) is estimated to range between 2% and 6%, though rates vary depending on the studied population, diagnostic practices, and follow-up duration [11]. Adherence to treatment and regular follow-up significantly reduces progression rates, with malignancy being virtually absent in patients who maintain consistent disease suppression. Conversely, irregular follow-up and treatment non-adherence, often due to socioeconomic barriers or lack of risk awareness, are strongly associated with higher rates of progression. Data further underscore that the cumulative probability of neoplastic progression increases with longer disease duration, reaching as high as 36.8% over 25 years. Therefore, early diagnosis, rigorous exclusion of dVIN and invasive foci, and lifelong management are paramount. The oncological pathway of LS, through chronic inflammation to dVIN and SCC, leaves no room for conservative approaches in refractory cases, necessitating the exploration of safe and effective second-line therapies while ensuring regular surveillance and disease stabilization.

Regarding the therapeutic approach, using high-potency TCS is the evidence-based therapy. In the acute phase, TCS promote the healing of fissures and erosions and reduce hyperkeratosis. In the chronic phase, they prevent structural changes and reduce the incidence of vSCC [10,11,12,13]. The most commonly used is clobetasol propionate 0.05%, followed by mometasone furoate, which is preferred in the Australasian management consensus due to its greater availability and safety. It is the first-choice therapy for children and pregnant women [11]. An ointment vehicle is preferred over cream because of its lower risk of causing contact dermatitis and better skin penetration. Dosing regimens vary depending on the patient; they may be prescribed, e.g., once daily for three months and then on alternate days for the second month. For maintenance, TCS are recommended once or twice per week. The British guidelines published in 2018 are the only ones that recommend an “as-required” or symptom-based approach; additionally, no alternative treatments besides topical ultra-potent corticosteroids have demonstrated efficacy in reducing the incidence of vulvar squamous cell carcinoma (VSCC) in patients with lichen sclerosus (LS). While various second-line therapies have been explored, their benefit remains confined to symptom relief rather than addressing the underlying risk of malignant progression. As such, the continuous and appropriate use of high-potency corticosteroids, which is the only evidence-based intervention capable of stabilizing disease activity and mitigating oncological risk, remains the cornerstone of LS management. This highlights the necessity of adherence to approved corticosteroid regimens to effectively reduce the burden of VSCC [12].

Second-line treatments have not demonstrated superiority over TCS, but their combination offers certain advantages. Topical calcineurin inhibitors (TCI)—pimecrolimus and tacrolimus—effectively control symptoms without corticosteroid side effects, making them suitable for alternating use [14]. Topical retinoids (e.g., tretinoin 0.025%, retinaldehyde 0.05%) can be added to TCS especially for hyperkeratosis [15]. Topical hormones are not advised for LS. Testosterone 2% has reduced symptoms in some trials, but side effects such as hirsutism, clitoral hypertrophy, acne, and amenorrhea contraindicate its use. Topical estrogens may be considered for vaginal dryness and lubrication in sexually active postmenopausal women, but they are not indicated as monotherapy for vLS [10,16]. A new treatment, MC2-25 cream, is being tested in a clinical trial. This di-peptide acts as an isocyanate scavenger, inactivating urea [17]. Increasing evidence suggests that the prolonged exposure of the genital epithelium to urine is a key contributor, particularly in men and older women with urinary incontinence (UI). The pattern of genital involvement and skin changes around urostomies further support this theory [18,19].

Systemic therapy is advised for refractory cases and those involving extensive body surface area. Methotrexate and systemic retinoids have demonstrated efficacy in a limited number of patients. In extragenital lichen sclerosus (eLS), promising results have been reported with dupilumab and secukinumab. The advent of biologics and Janus kinase (JAK) inhibitors is anticipated to yield further insights in the coming years. Surgical intervention is reserved for the management of vulvar squamous cell carcinoma (vSCC) and the reconstruction of vulvar anatomy, with circumcision in males being the only procedure with curative intent [20]. Given the high recurrence rates (56%), surgical approaches should be restricted to stable cases and supplemented with postoperative topical immunosuppressive therapy. Vulvar regenerative procedures, including autologous lipotransfer and platelet-rich plasma (PRP), may be employed to restore tissue volume and mitigate fibrosis. A multidisciplinary approach combining pelvic floor physiotherapy and sexual therapy is also recommended [21,22].

At present, laser therapy does not have sufficient evidence to support its use as a standard treatment for vulvar lichen sclerosus (vLS). Nonetheless, clinical trials and observational studies have reported favorable outcomes in terms of symptom relief and quality-of-life improvement, suggesting its potential as an adjunctive therapy. The fractional carbon dioxide laser (FxCO_2_) is the most extensively studied modality. Operating at a wavelength of 10,600 nm, it targets intracellular water, resulting in selective heating and tissue vaporization, which produces superficial microabrasions. In fractionated mode, energy is delivered in a columnar pattern, alternating between treated and untreated areas, facilitating a shorter recovery period. At the dermal level, this technique induces collagen matrix degradation, stimulates new collagen synthesis, and promotes neovascularization, thereby enhancing tissue repair and remodeling.

To further explore these potential benefits, we conducted a study at our center involving women referred for vulvar lichen sclerosus (vLS) [2,3].

- The primary objective was to assess the efficacy of this treatment in controlling symptoms and its impact on patients’ psychosocial and sexual well-being.

A secondary objective was to identify prognostic factors or variables associated with a more favorable treatment response.

## 2. Materials and Methods

### 2.1. Study Design

A single-center, prospective observational study was conducted in the Laser Department of the San Cecilio University Hospital, Granada, Spain. The data collection period took place between January 2022 and February 2024. The sampling was consecutive for those patients who met the inclusion criteria and agreed to participate in the study.

### 2.2. Inclusion and Exclusion Criteria

The study only included adult female patients (over 18 years of age) with a clinical and/or histological diagnosis of vLS who understood the study and agreed to participate. Patients had been undergoing refractory to high-potency topical corticosteroid treatment for a minimum period of six months. Patients with dermatological, psychiatric, or systemic diseases that could affect study outcomes, as well as those who declined to sign informed consent forms, were excluded from the study.

### 2.3. Variables 

Data on epidemiological characteristics, clinical variables, and biometric parameters were collected through structured clinical interviews. The diagnosis of LS was clinically established using the Clinical Score System in the vLS tool (CSS-VLS), with a score > 4 required for confirmation [23]. If there was diagnostic uncertainty, a confirmatory biopsy was required. 

Patients rated symptoms (itching, pain, and dyspareunia) on a 0–10 visual analogue scale (VAS) scale at baseline and at the end of follow-up. 

The psychosocial and sexual variables were collected by means of questionnaires validated in Spanish.

(a) Dermatology Life Quality Index (DLQI): A dermatology-specific questionnaire composed of 10 items assessing the impact of skin conditions on patients’ quality of life. The areas considered include symptoms and feelings, daily activities, leisure, work or school, personal relationships, and treatment. Each item is rated on a four-point scale: 0 (“Not at all”, no impact), 1 (“A little”), 2 (“Quite a lot”), and 3 (“Very much”, significant impact). The total score ranges from 0 to 30, with higher scores indicating a greater negative impact on quality of life, categorized from “no effect” (0–1) to “very large effect” (21–30) [24].
(b) Female Sexual Function Index (FSFI): A validated 19-item questionnaire that assesses different aspects of female sexual function over the past four weeks, including desire, arousal, lubrication, orgasm, satisfaction, and pain. Each item is rated on a scale from 0 to 5, with lower scores indicating greater sexual dysfunction. The total score is derived by summing subscale scores, ranging from 2 to 36; lower values reflect greater impairment in sexual function [25].
(c) Beck Depression Inventory, Second Edition (BDI-2): a widely used tool to assess the severity of depressive symptoms. It consists of 21 items, each scored from 0 to 3, with higher scores representing more severe depressive symptoms. Total scores are interpreted across ranges, from “minimal” (0–13) to “severe depression” (29–63) [26].

These instruments were chosen due to their validation and frequent use in clinical research, ensuring the reliability and relevance of the data obtained concerning quality of life, sexual function, and emotional state in patients. 

Finally, the study also included the Patient Global Impression of Improvement (PGI-I), a scale for patients to rate their overall improvement after treatment. The PGI-I is a single-item measure that categorizes patient response on a 7-point scale, ranging from “very much improved” (1 point) to “very much worse”, (7 points) providing a global assessment of treatment effectiveness from the patient’s perspective [27].

### 2.4. Treatment Protocol

**Figure 2:**
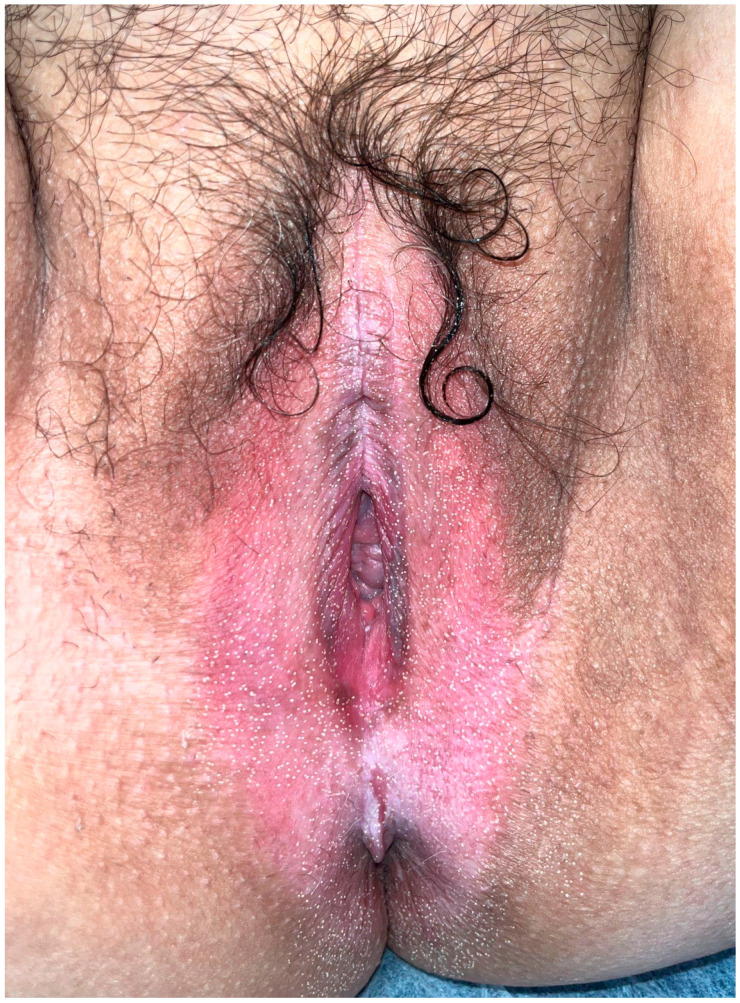
FxCO_2_ endpoint. Small white dots and discrete oedema and erythema. Ablative fractional lasers produce no specific therapeutic endpoints. The size of the holes depends on the device. Bleeding should not appear; this may indicate relative depth of procedure [28].

A protocol was designed consisting of four FxCO_2_ laser sessions (Silklase^TM^ Ent Co_2,_ INTERmedic S.A., Barcelona, Spain), administered at three-month intervals. Before the procedure, a generous amount of topical anesthetic (lidocaine/prilocaine 2.5%) was applied under occlusion for 30 min. Then, the area was disinfected and the session was performed with standard parameters (power 17 W, pulse duration 3.5 ms, energy density 209.9 J/cm^2^, square spot size 10 × 47 mm). Post-laser care included applying an antibiotic cream (mupirocin) twice daily for the first five days. Afterward, patients returned to their usual corticosteroid routine: daily use for one month during flare-ups, then twice weekly for maintenance. Patients who reported significant improvement and wished to continue treatment were offered additional laser sessions, scheduled at more extended intervals (Figure 2). These sessions were not recorded in this study. If patients had a history of genital herpes, prophylactic treatment with valacyclovir 500 mg every 12 h was prescribed for the following 5 days.

### 2.5. Statistical Analyses

The continuous variables were summarized using means and standard deviation to provide a general description of the sample characteristics. Categorical variables were described using frequencies and percentages.

The paired Student’s *t*-test was applied to evaluate differences in scale scores between baseline and post-treatment, allowing for comparison within the same subjects to identify statistically significant changes attributable to the intervention. To determine the relationship between improvement after treatment (measured using the different VAS scales and questionnaires of quality of life, depression, and sexual function) and the main demographic and disease variables (age, Body Mass Index, smoking, nulliparity, sexual activity, years of evolution, and pre-treatment physical examination), linear regression models were conducted.

The level of statistical significance for all tests was set at *p* < 0.05. All statistical analyses were conducted using Stata software (version 16.1, Stata Corp., College Station, TX, USA).

### 2.6. Ethics

The research was approved by the Hospital Universitario San Cecilio Ethics Committee (HUSC-003-2023, 20 May 2023) and conducted in accordance with the principles set forth in the Declaration of Helsinki.

## 3. Results

From January 2022 to February 2024, a total of 75 women with a clinical and/or histological diagnosis of LS were enrolled in the study, with a mean disease duration at a baseline of 6.5 years. Thirty-seven patients (49.3%) underwent confirmatory biopsy. 

### 3.1. Univariate Analysis

The demographic characteristics of the study population are summarized in Table 1. The majority of participants were postmenopausal (*n* = 67). Hypertension was the most prevalent comorbidity (*n* = 28), followed by dyslipidemia (*n* = 19) and hypothyroidism (*n* = 16). Nine patients reported additional dermatoses or autoimmune conditions, including allergic contact dermatitis, lichen planus, Sjögren’s syndrome, psoriasis, alopecia areata, frontal fibrosing alopecia, vitiligo, Behçet’s syndrome, and cicatricial pemphigoid. A history of gynecological surgery was noted in twenty patients (26.7%), although only two of these procedures were related to complications of lichen sclerosus (synechiae).

Based on clinical evaluation, whitish atrophic plaques were observed in 53 patients (70.7%), fissures and erosions were present in 34 subjects (45.3%), and 31 (42.3%) showed perianal involvement. In terms of sequelae, 59 (78.7%) presented with resorption and/or labial fusion, and 42 (56.0%) had narrowing of the introitus. Extragenital affectation was observed in 10 patients (13.3%).

Sixty-four women completed four laser sessions and a 3-month follow-up. Reasons for dropping out were difficulty attending appointments (3), perceived lack of effectiveness (3), illness (2), unrelated death (1), pregnancy (1), and unknown reasons (1). No adverse effects were reported with the treatment, except for mild discomfort in the days following the laser procedure. 

### 3.2. Bivariate Analysis

In the 64 women who completed the study, all scale scores improved following the laser treatment (Table 2). Symptom response demonstrated a significant reduction in severity across several areas: itching (7.53 ± 3.02 versus 4.08 ± 3.07), pain (5.83 ± 3.84 versus 2.42 ± 2.85), and dyspareunia (8.26 ± 2.82 versus 6.34 ± 3.30) were statistically significant. In addition, of the 38 patients (50.7%) who were sexually active at baseline, 23 (60.5%) experienced a decrease in pain during sexual intercourse after treatment.

A total of 10 patients refused to complete questionnaires on quality of life, depression, or sexual function. In the other 54 women, DLQI scale scores decreased significantly from 10.72 ± 7.25 to 5.94 ± 5.16 (*p* < 0.001), along with BDI scores from 16.66 ± 12.64 to 5.94 ± 5.16 (*p* < 0.001). In addition, FSFI scale scores improved significantly (10.48 ± 8.46 versus 15.52 ± 9.59; *p* < 0.001). The results of subjective improvement among the 64 patients who completed the study, measured using the PGI-I scale, showed high satisfaction with the treatment. Sixty patients rated their improvement between 1 (“very much better”) and 3 (“a little better”), with a median score of 2.2, indicating much improvement. Three patients reported “no changes” and only one patient reported being worse after the laser treatment. 

A total of 10 patients refused to complete questionnaires on quality of life, depression, or sexual function. In the other 54 women, DLQI scale scores decreased significantly from 10.

When analyzing the improvement experienced after laser treatment according to different demographic and disease variables, a statistically significant association was only found for a greater improvement in FSFI in women who had erosions or fissures before treatment compared to women without erosions or fissures.

## 4. Discussion

The findings of this study highlight the potential of fractional CO_2_ (FxCO_2_) laser therapy as an effective alternative for managing symptoms and enhancing quality of life in women with vulvar lichen sclerosus (vLS) that is refractory to treatment with topical corticosteroids (TCS). By addressing both physical symptoms and psychosocial impacts, this intervention offers a promising adjunctive option for patients who do not achieve adequate relief with conventional therapies. These results are discussed in the context of published evidence, further exploring the role of laser-based treatments in the comprehensive management of vLS. Our study population exhibited characteristics comparable to those of the general population affected by lichen sclerosus (LS). The majority of participants were postmenopausal (89.3%), with a mean BMI of 27.3, and common comorbidities such as hypertension (37.3%), dyslipidemia (25.3%), and hypothyroidism (21.3%). Additionally, some participants presented with concomitant autoimmune conditions, including alopecia areata, vitiligo, and Sjögren’s syndrome. Known risk factors for LS, such as obesity (mean BMI 27.3), multiparity (average of two children), and prior gynecological surgeries (26.7%), were prevalent in the cohort [4].

Clinically, a notable proportion of patients exhibited aesthetic sequelae, including labial fusion (78.7%) and introitus narrowing (56%). The average disease duration of 6.5 ± 5.9 years does not appear sufficient to explain these sequelae. This discrepancy may be attributed to an underestimated disease duration, potentially due to a high percentage of asymptomatic women in the early stages of LS, as suggested by reports that indicating up to one-third of patients may be asymptomatic [10].

Only one patient (0.01%) had a history of vulvar squamous cell carcinoma (vSCC), a rate lower than the expected 4–6%, likely reflecting the relatively younger mean age of the sample (60.83 years). Extragenital involvement was observed in 13.3% of participants, aligning with the reported prevalence of 15–20% in the general population. Overall, this study group is representative of and relevant for evaluating treatment efficacy and outcomes in typical LS patients.

Symptom relief was significant after four FxCO_2_ laser sessions. VAS scores decreased for itching, pain, and dyspareunia. These findings are consistent with the broader literature. Ogrinc et al. conducted a randomized controlled trial (RCT) using three Nd:YAG laser sessions and found a symptomatic improvement in the laser-treated group (LG) compared to the corticosteroid-only group (CG) at 1–3 months of follow-up. The mean VAS score decreased for burning (3.3 points, *p* = 0.001), for itching (4.1 points, *p* < 0.001), and for pain (3.3 points, *p* = 0.001) [29]. Similarly, another prospective longitudinal study with two cycles of FxCO_2_ laser treatment reported significant VAS improvements in vulvar itching (6 pts, *p* < 0.001), vulvar dryness (5 pts, *p* < 0.001), superficial dyspareunia (6 pts, *p* < 0.001), and sensitivity during intercourse (4 pts, *p* < 0.001). However, vulvodynia did not show a significant change following treatment [30]. 

Our findings demonstrate that fractional CO_2_ (FxCO_2_) laser therapy significantly enhances quality of life (QoL), as well as psychological and sexual well-being. Specifically addressing sexual function, among the 38 patients (50.7%) who were sexually active at baseline, 23 (60.5%) reported reduced pain during intercourse, with a mean improvement in Female Sexual Function Index (FSFI) scores of 5.04 points at six months post-treatment (*p* = 0.006). Similarly, an Italian study reported FSFI improvements in 55% (6/11) of patients three months after FxCO_2_ therapy, with a mean score increase of 4.5 points at six-month follow-up compared to baseline (*p* < 0.05). Notably, in that study, postmenopausal women underwent FxCO_2_ treatment for both the vaginal canal, using an intravaginal device, and the external vulvar regions. This combined approach is particularly relevant given the high prevalence of vulvar lichen sclerosus (VLS) in postmenopausal women, who are also susceptible to vaginal atrophy secondary to hypoestrogenism [31]. In our study, patient perception of improvement was notably high, with 60 out of 64 patients reporting some degree of improvement. In a randomized controlled trial (RCT), Burkett et al. [32] found that 89% of patients in the CO_2_ laser group (LG) rated their improvement as “better” or “much better” on the Patient Global Impression of Improvement (PGI-I) scale, compared to 62% in the control group (CG), although the difference was not statistically significant (*p* = 0.07). Satisfaction, as measured by the Patient Global Impression of Severity (PGI-S), was significantly higher in the LG than in the CG (81% vs. 41%, *p* = 0.01). In a continuation of this study, a crossover trial with non-responding patients from both groups demonstrated a marked increase in satisfaction when patients were switched to the LG (22% in CG vs. 52% in CG→LG, *p* = 0.001) [33]. These outcomes may be influenced by increased medical attention or the introduction of a new therapeutic option. Many of the women in our study had previously been treated with topical corticosteroids (TCS) over extended periods with limited success, which often led to decreased motivation, poor adherence to treatment, and suboptimal disease control. The addition of novel therapies to standard treatment protocols appears crucial for maintaining therapeutic adherence and improving patients’ perception of their condition.

Histological comparison was not performed due to the absence of baseline biopsy data for approximately 50% of patients. Published results regarding histological changes after laser treatment are inconsistent. Some studies have reported no significant histological differences between CO_2_ laser and TCS or between low-energy laser and sham laser treatments, while others have noted reductions in basement membrane thickness, a decrease in the dermal hyalinized zone, and a reduction in inflammatory infiltration following laser treatment [34,35,36,37,38].

Clinical signs were not assessed post-treatment, as no validated tools exist for scoring the severity of lichen sclerosus (LS). Sheinis and Shelk [39], in a Delphi Consensus, proposed a 24-item severity scale, including categories such as symptoms, signs, and architectural changes. This proposal has received support, with Stewart et al. [31] designing a 4-point scoring system in a single-arm trial to assess these items. Using this scale, they observed clinical improvement in 80% of patients over 12 months, with reductions in whitening, parchment-like skin, labial fusion, narrowing of the introitus, and sclerosis. Krause et al. [40] used the Clinical Severity Score for Vulvar Lichen Sclerosus (CSS-VLS) to measure pre- and post-treatment changes but found no significant differences between the LG and sham laser groups. Other proposed scales, such as the Clinical Lichen Sclerosus Score (CLISSCO) and the Vulvar Architecture Severity Scale (VASS), have yet to gain widespread acceptance, and interobserver variability remains a potential limitation [41,42,43].

The optimal number and timing of laser sessions have not been standardized. Published studies recommend two to five sessions spaced 2 to 6 weeks apart. In our study, sessions were spaced at 12-week intervals, achieving comparable results. As laser treatment is an expensive, privately funded procedure, extending the time between sessions may enhance cost-effectiveness. The duration of laser response remains uncertain, although efficacy appears to decline approximately one year after treatment. No significant differences in quality of life (QoL) or most clinical vulvar changes were observed between the LG and CG after 12 months, and treatment satisfaction also decreased over the year. Additionally, the frequency of corticosteroid use increased to an average of 2.06 times per week at 12 months. Further research is needed to determine the optimal number of sessions and to investigate whether annual maintenance treatments are necessary to sustain the benefits of laser therapy.

## 5. Conclusions

In conclusion, this study underscores the positive and promising effects of the FxCO2 laser in alleviating symptoms and enhancing quality of life, including sexual well-being, in patients with lichen sclerosus (LS). It demonstrates that the FxCO_2_ laser is a valuable and well tolerated therapeutic option, particularly when combined with topical corticosteroids (TCS). Spacing treatment sessions at 12-week intervals produces outcomes comparable to those reported in previous studies, while also offering a more cost-effective approach. However, the relatively short follow-up period limits the ability to assess the long-term effects of treatment and to determine whether additional cycles of therapy may yield sustained benefits.

## 6. Limitations 

The limitations of this study include its non-randomized design, a relatively small sample size, and the absence of an untreated control group or sham laser group. Additionally, the study is subject to selection bias due to consecutive sampling, as patients referred to the laser unit typically present with more advanced disease. Another limitation is the reliance on subjective symptom scores, which may be influenced by a placebo effect, as all participants were aware of receiving active treatment. Furthermore, questionnaire responses may have been biased by symptom flare-ups at the time of completion. The lack of comparison between objective diagnostic methods before and after treatment represents another limitation, as such a comparison could have provided a more comprehensive evaluation of therapeutic outcomes. Although histopathological examination was referenced, less invasive alternatives, such as high-frequency ultrasound (HFUS), have shown promise in the evaluation and monitoring of vulvar lichen sclerosus (VLS) and may offer a non-invasive tool to track structural changes over time. Lastly, a significant proportion of the sample consisted of elderly individuals who were not sexually active, which may limit the generalizability of the findings regarding sexual function.

## Figures and Tables

**Table 1:** Demographic and clinical characteristics of the patients at baseline.

Variable	Total (*n* = 75)
Age (years), mean ± SD	60.83 ± 11.25
Range	32–82
BMI (kg/m^2^), mean ± SD	27.3 ± 5.18
Parity, median (IQR)	2 (1)
Smoking habit, *n* (%)	
Current smoker	9 (12.0)
Past smoker	9 (12.0)
Never smoker	57 (76.0)
Course of disease (years), mean ± SD	6.5 ± 5.9
Previous diagnosis of VSCC, *n* (%)	1 (1.3)
Sexually active, *n* (%)	43 (57.3)
Prior TCIs treatment, *n* (%)	22 (29.3)
Menopause, *n* (%)	67 (89.3)
Hormone replacement therapy, *n* (%)	8 (10.7)
Gynecological surgery	20 (26.7)
Comorbidities, *n* (%)	28 (37.3)
Hypertension	19 (25.3)
Dyslipidemia	9 (12.0)
Diabetes	16 (21.33)
Hypothyroidism	3 (4.0)
Vitamin D deficiency	5 (6.7)
Respiratory disorders	2 (2.7)
Gastrointestinal disorders	2. (2.7)
Migraine	4 (5.3)
Malignant diseases	9 (12.0)
Other dermatoses	20 (26.7)

SD, standard deviation. BMI, Body Mass Index. IQR, interquartile range. VSCC, vulvar squamous cell carcinoma. TCIs, topical calcineurin inhibitors.

**Table 2:** Comparison of outcome measures between baseline and after 4 sessions of CO_2_.

Scale	Baseline	After Laser	Effect Size (95% CI *)	*p* Value
VAS itching (*n* = 64)	7.53 ± 3.02	4.08 ± 3.07	3.45 (2.43–4.47)	<0.001
VAS pain (*n* = 64)	5.83 ± 3.84	2.42 ± 2.85	3.41 (2.49–4.32)	<0.001
VAS dyspareunia (*n* = 38)	8.26 ± 2.82	6.34 ± 3.30	1.92 (0.92–2.92)	<0.001
DLQI (*n* = 54)	10.72 ± 7.25	5.94 ± 5.16	4.78 (3.01–6.54)	<0.001
BDI (*n* = 54)	16.66 ± 12.64	11.67 ± 10.84	5.00 (3.09–6.90)	<0.001
FSFI (*n* = 32)	10.48 ± 8.46	15.52 ± 9.59	−5.04 (−8.00–−2.07)	0.006

* CI, confidence interval. VAS, visual analogue scale. DLQI, Dermatology Life Quality Index. BDI, Beck Depression Inventory. FSFI, Female Sexual Function Index.

## Data Availability

The original contributions presented in this study are included in the article. Further inquiries can be directed to the corresponding author.
